# Study of Different Personalised Dietary Plans on Eating Behaviour, Body Image and Mood in Young Female Professional Handball Players: A Randomised Controlled Trial

**DOI:** 10.3390/children10020259

**Published:** 2023-01-31

**Authors:** Laura Miralles-Amorós, Manuel Vicente-Martínez, María Martínez-Olcina, Nuria Asencio-Mas, Lucía Gonzálvez-Alvarado, Marcelo Peñaranda-Moraga, Belén Leyva-Vela, Rodrigo Yáñez-Sepúlveda, Guillermo Cortés-Roco, Alejandro Martínez-Rodríguez

**Affiliations:** 1 Department of Analytical Chemistry, Nutrition and Food Science, University of Alicante, 03690 Alicante, Spain; 2 Faculty of Health Science, Miguel de Cervantes European University, 47012 Valladolid, Spain; 3 Department of Health, Vinalopó University Hospital, 03293 Elche, Spain; 4 Faculty of Education and Social Sciences, Universidad Andres Bello, Viña del Mar 2520000, Chile; 5 School of Education, Pedagogy in Physical Education, Sports Coach, Universidad Viña del Mar, Viña del Mar 2520000, Chile; 6 Alicante Institute for Health and Biomedical Research (ISABIAL), 03010 Alicante, Spain

**Keywords:** eating disorders, body image, mood state, young adult, handball, low energy availability

## Abstract

Low energy availability may precede or be caused by cognitive disturbances in professional athletes. Related psychological problems include disordered eating patterns, body shape preoccupation, depression or anxiety. The objective of this research was to evaluate the effects of different personalised dietary plans on psychological factors in young professional female handball players with low energy availability. This 12-week randomised clinical trial involved 21 female players aged 22 ± 4 years, 172.0 ± 5.4 cm and 68.4 ± 6.7 kg divided into three groups (FD: free diet; MD: Mediterranean diet; HAD: high antioxidant diet). Eating behaviour (Eating Attitude Test, EAT-26: diet, bulimia and oral control subscales), body image (Body Shape Questionnaire, BSQ) and mood state (Profile of Mode State, POMS: tension, vigour, anger, depression, fatigue) were assessed. All participants showed low energy availability (<30 kcal/lean mass per day). The different plans showed no significant differences between them but significant differences over time within groups for the variables: body image, Tension, Vigour and Depression (*p* < 0.05). Eating behaviour improved slightly but did not show statistically significant changes. Following an adequate nutritional planning for athletes seems to improve the mood and body perception of young female handball players. A longer intervention period is required to assess the differences between diets and improvement of other parameters.

## 1. Introduction

Professional athletes often suffer from energy deficits due to the long hours and intensity of training combined with strict diets for maximum performance. Low energy availability is related to both physiological and psychological disturbances [1]. Psychological problems can include disordered eating patterns, such as obsession with food and restriction or strict control of intake at meals, eating disorders (ED), preoccupation with body shape, depression, irrational behaviour, mood swings, social isolation or a severe level of anxiety [1]. These may precede or be caused by low energy availability [2].

Handball is a team sport in which the movements require a high level of coordination and the rhythm of the game is very fast [3], with high precision [4,5,6,7,8]. Certain players are expected to have a particular level of physical strength and body mass index (BMI), as is the case for the pivot, as a handball player must play between defenders, fight for position and create a throw-in [4,9]. 

The search for that optimal BMI often leads athletes to alter their eating habits and run the risk of developing ED [10]. EDs are serious mental illnesses, with high mortality rates, characterised by persistent disturbance in eating or eating-related behaviour causing significant impairment in health and psychosocial functioning [11,12].

The severity of the symptomatology associated with EDs stands out, coupled with strong opposition to treatment, a strong risk of recurrence of EDs and a significant degree of co-morbidity or emergence of new disorders such as addictions, depressive states, low self-esteem and problems with social behaviours. Among the factors that are related to the onset of EDs are: dieting, being female, being an adolescent, perfectionist behaviour, compulsivity, high expectations and having a distorted perception of body image [13].

Regarding the prevalence in athletes, most studies report a higher prevalence of ED in sports (13.5%) than in the general population (8.4–2.2%). However, no risk factors are found to be different from the general population, except for public exposure of the body and pressure from coaches [14].

Sports can be classified as lean or non-lean [15]. On the one hand, lean sports focus on low body weight for performance enhancement (e.g., dance, judo, long-distance running, swimming …) [16,17]. On the other hand, the non-lean ones do not focus on low body weight for performance; among these sports are team sports, golf, horse riding… [18]. The weight requirement in lean sports increases the risk of ED, as it leads athletes to engage in harmful behaviour to achieve a low weight [19]. However, although research is scarce, an elevated prevalence of ED is also found in non-lean team sports.

In men’s team sports, Baldó Vela et al. [20] found that 18.5% of the population presented a clinical picture compatible with a diagnosis of ED. Moreover, research conducted on the Spanish beach handball national team players found a prevalence of ED of 11% in women and 3% in men [21]. No research has analysed the prevalence of ED in young professional beach handball players.

These diseases are closely related to body image, as it has been observed that team sports players also report dissatisfaction in certain body areas, with body mass index being a significant mediator of their perceived physical ability. Morano et al. [22] agree that personal, psychological and physical factors need to be focused on to achieve efficiency in all sports and weight groups [22]. 

Diet is a crucial variable for a healthy life. An increasing number of studies in recent years support the hypothesis that a proper diet has numerous effects at the organ level. In a recent systematic review, it has been observed that a balanced and healthy diet, supported by the guidelines of the Mediterranean Diet, shows improvements not only on cardiovascular and obesity parameters, but also on brain function and mood [23].

In the approach to EDs and their symptoms associated with body image and mood, nutritional rehabilitation and weight normalisation is a central goal in the early stages of therapy [24]. Recovery rates are higher than 40% in those cases in which nutritional treatment is included alongside classic psychotherapy [25].

In another intervention carried out by Hart et al. [5], a more rapid reduction of symptoms associated with ED, especially bulimic behaviours, together with an improvement in depressive symptoms, was observed with nutritional intervention [5].

The Mediterranean diet is a healthy eating pattern associated with a reduced risk of chronic diseases [26,27,28]. This dietary pattern is characterised by a high consumption of plant foods, cereals, legumes, fish and olive oil as the main source of fat. The biological mechanisms behind this disease prevention are the high amounts of polyphenols, dietary fibre, low glycaemic index, antioxidants and other compounds such as monounsaturated and polyunsaturated fatty acids found largely in leafy vegetables, fruits and olive oil [29,30,31]. Further evidence has supported the relationship between the Mediterranean diet and depression, showing a potential protective role against the onset of depressive symptoms [32,33]. 

According to research by Bonaccio et al. [34], monounsaturated fatty acids, whose main source is olive oil, and dietary fibre were positively associated with mental and physical health. Similarly, higher intake of dietary antioxidants represented better mental fitness; however, dietary antioxidant levels were not associated with physical health and therefore could not explain the observed relationship between higher adherence to the Mediterranean diet and better self-reported physical fitness [34]. 

Other previous studies have investigated the role of the basic components of the Mediterranean diet on quality of life and found direct associations between some of its components, such as fish consumption [35]. Inverse associations were also found between fruit and vegetable consumption and the incidence of depressive symptoms [36]. 

Positive associations between antioxidant intake and cognitive function have been extensively documented [37,38] and were mainly attributed to the preventive and retarding activity of antioxidants on free radical production [39]. Oxidative stress has been implicated in the pathophysiology of many neuropsychiatric disorders, such as schizophrenia, bipolar disorder and major depression [40,41,42]. However, such an approach does not consider the diet as a whole, which probably ends up underestimating the interactions between various foods and nutrients [43]. 

Although previous studies have provided interesting data on the direct association between the Mediterranean diet and a high consumption of fruit and vegetables on quality of life, other related parameters such as body image have so far not been addressed.

The principal hypothesis of this research is that professional female handball players who follow dietary guidelines based on a Mediterranean diet will improve their eating behaviours, mood and body image.

The primary objective of this investigation is to evaluate the impact of different personalised nutritional plans in professional female handball players with energy deficits on eating disorders, mood and body image. The secondary objective is to assess whether a complete dietary pattern (Mediterranean diet) improves eating behaviours, body image and mood more than a focus on antioxidant nutrients alone.

## 2. Materials and Methods

### 2.1. Study Design

A randomised controlled trial was developed over 12 weeks. The study participants represent the elite of women’s handball, as they are the winners of the Spanish Supercopa and Copa de la Reina.

The guidelines of the CONSORT (CONsolidated Standards of Reporting Trials) 2010, Declaration of Helsinki and the EEC Good Clinical Practice recommendations (July 1990 Document 111/3976/88) were followed throughout the research. In addition, players filled out informed consent forms prior to the start of the research, approved by the Ethics Committee of the University of Alicante (Spain) (UA-2021-03-11, 17 June 2021) and all personal information was coded in order to maintain the confidentiality of all participants. The research was submitted to the official clinical trials database ClinicalTrials.gov, under registration number NCT05567211.

### 2.2. Participants

The study involved 21 young adult female professional handball players. All of them performed 12 h of training per week plus matches. Different exclusion criteria were considered: refusal to sign the informed consent, medication influencing the results and presence of chronic diseases. However, all players were included in the study.

In accordance with published recommendations [44], an online computer software was used to electronically randomise subjects using a block design in three arms (one control and two experimental). A researcher external to this study was in charge of preparing these envelopes.

### 2.3. Data Collection

#### 2.3.1. Dietary Intake

Dietary intake was recorded by means of a 7-day self-record where all weighed foods, liquids ingested and times of the different intakes were to be indicated. Supplements taken and pre-, during and post-training intakes were also to be included. From this record, quantitative and qualitative evaluations were carried out with the software Dietopro.com [45]. This software provided information on energy, macronutrients (carbohydrates, proteins, lipids, fibre) and micronutrients (vitamins and minerals) consumed during the different periods. Players were trained prior to self-completion of the questionnaire to quantify all foods and beverages consumed and were provided with photos and instructional videos to promote accurate self-reporting, with no ingredients overlooked and with information on food weight and quantity consumed. Daily intakes collected over 7 consecutive days at the three time points were averaged, obtaining both absolute, percentage and per kg weight per day values.

#### 2.3.2. Body Composition

Weight and height variables were measured for each subject. The standard protocol of the International Society for the Advancement of Kineanthropometry (ISAK) [46] was followed.

All determinations were carried out by the same researcher and under the same conditions: early in the day, fasting and under basal conditions, with the same equipment and at room temperature (22 ± 1 °C). The time of the menstrual cycle was taken into account so that they were always in the same phase.

Height was measured with the athlete’s head following the Frankfort plane with a millimetre-accurate mobile stadiometer (Seca 213, SECA Deutschland, Hamburg, Germany). Weight was recorded with as little clothing as possible with a 100 g precision digital scale (BC545N, Tanita, Tokyo, Japan). Body mass index (BMI) was obtained from this dates and classified according to World Health Organisation standards [47].

#### 2.3.3. Eating behaviour

The Eating Attitudes Test, in its reduced 26-item version (EAT-26) [48], was used in a self-administered manner. This questionnaire has been validated in Spanish women [49] as a possible screening for the future presence of an ED. The cut-off point for determining the possible presence of ED is 20 points, with a Likert-type scale where 1 is never and 6 is always [50]. All questions are scored as “0, 0, 0, 0, 1, 2 and 3” except item 25, where the score is reversed. This total score is further divided into three subscales: food restriction; bulimia and preoccupation with food; and oral control [51].

#### 2.3.4. Body Shape

The Body Shape Questionnaire (BSQ) [52] was used to assess body dissatisfaction, fear of gaining weight, desire to lose weight, feelings of low self-esteem about physical appearance and avoidance of situations involving exposure to the physique in a self-administered manner. This version has been validated and previously used in female athletes by Evans and Dolan [53]. The questionnaire consists of 14 items that are scored on a 6-point Likert scale: “never”, “rarely”, “sometimes”, “often”, “very often” and “always”. The score ranges from 14 to 84 points with the values being those that are related to a worse body image.

#### 2.3.5. Mood state

The Profile of Mood States (POMS) questionnaire in its abbreviated self-administered version was used to assess mood [37]. It consists of 30 items rated on a 5-point Likert-type scale: 0 = “not at all” and 4 = “very much”. This scale provides information on five factors: anger, fatigue, vigour, tension and depressed state. The stress, anger, depression and fatigue factors provide information on negative theoretical components, while the vigour factor provides information on positive theoretical components.

### 2.4. Intervention

A personalised dietary plan was made for each of the participants and followed for 12 weeks. Three groups of seven randomly distributed players were established: the control group that followed a free diet with healthy lifestyle recommendations for sportswomen (FD); the experimental group Mediterranean Diet (MD); and the experimental group high-antioxidant diet (HAD). The software used in the development of the diet was Dietopro (Dietopro, Valencia, Spain) [54]. The plans were structured identically so that the players could not identify how their diet differed. 

Energy expenditure was calculated from the basal energy expenditure calculated using the Harris–Benedict formula and multiplied by the EEE and the thermic effect of food. Daily physical activities and the handball-specific EEE were estimated using METs and their equations [55].All planning was done according to the recommendations of professional female handball players [56].

A total of 7 g/kg/day of carbohydrate (50–55% of the total caloric value), 1.5 g/kg/day of protein (15–17% of the total caloric value) and 30% of the total caloric value for fat were provided. 

The MD was based on whole grains, fish, white meat, pulses, vegetables, olive oil and Mediterranean fruits such as oranges, mandarins, grapes, pineapple, bananas, apples and pears. This pattern met the recommended daily allowances (RDAs) for micronutrients and 100% of the RDAs for the antioxidants studied (alpha-tocopherol, vitamin C and vitamin A) [57]. The HAD prioritised the presence of fruits high in antioxidants, such as blueberries, beetroot and pomegranate, providing 200% of the RDA for alpha-tocopherol (30 mg), 500% of the RDA for vitamin C (450 mg) and 200% of the RDA for vitamin A (1800 μg) [58].

The players were contacted weekly by telephone to improve compliance with the nutritional advice and were provided with a list of equivalents for them to finalise and adapt to their dietary availability.

### 2.5. Statistical Analysis

Jamovi 1.1.3.0 software (The jamovi project, Sydney, Australia) was used to perform the statistical analyses. Descriptive statistics were calculated for all variables (mean ± standard deviation) and the normality of the distribution was tested using the Shapiro–Wilk test. For equality of variances, Levene’s test was performed, and analysis of covariance (ANCOVA) (general linear model; time × group) with BMI as a covariate was applied to analyse the effects of the intervention on the assessments. For time-by-group interaction effects, partial eta squared effect sizes (η^2^_p_) were calculated (η^2^_p_ ≥ 0.01 indicates a small effect, ≥0.059 a medium effect, and ≥0.138 a high effect). If significant main effects were found, post hoc tests (Tukey and Bonferroni) were performed. The level of statistical significance was set at *p* < 0.05. In addition, to establish connections between study variables, Pearson’s correlation test was used on correlations to determine effect sizes [43,44], with 95% confidence intervals.

## 3. Results

**Figure 1:**
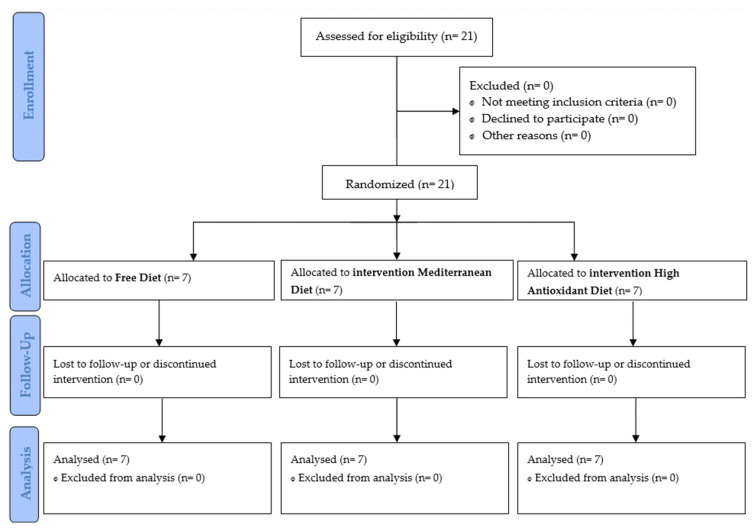
Consort Flow Diagram.

The study involved 21 young female professional handball players with a mean age of 22 ± 4 years divided into three groups (FD: free diet; MD: Mediterranean diet; HAD: diet high in antioxidants) (Figure 1). Recruitment took place during the pre-season 2022, and the intervention was conducted for 12 weeks, starting in August (PRE), with a middle measurement (PER) and a final measurement in November (POST).

All the players had an energy intake of less than 30 kcal/kg lean body mass per day, so they had a low energy availability and risk of RED-S. No significant differences in confounding variables were found either between the groups or during the intervention. Baseline data were collected as shown in Table 1.

Psychological variables related to eating behaviour (Table 2), body image and mood were analysed (Table 3). 

For eating behaviour, in the subscales diet, bulimia and oral control and individual items no significant differences were observed over time or between intervention groups (*p* ≥ 0.05).

For body image, significant differences were found in the post hoc analyses without correction of the MD group between PRE and POST times (*p* = 0.049), decreasing the score obtained in the BSQ questionnaire. However, in the Tukey and Bonferroni post hoc analyses, no statistically significant differences were detected (*p* = 0.526 and *p* = 1.000, respectively).

Concerning the analysis of the different mood states, no significant differences were found in the Vigour and Fatigue scales. 

Significant differences were found in the Tension scale in the HAD group between the PER and POST measurement in the post hoc analyses without correlation (*p* = 0.045) with a lower score after the intervention. However, no statistically significant differences were detected in the Tukey and Bonferroni post hoc tests (*p* = 0.499 and 1.000, respectively).

In the Anger scale, significant differences were found in the post hoc analyses in the MD group between PER and POST (*p* = 0.021) and in the HAD group between PRE and PER (*p* = 0.049), and PER and POST (*p* = 0.007) times, improving mood with lower scores. However, all groups gave results higher than *p* = 0.05 for Tukey’s and Bonferroni’s post hoc analyses.

The results of the Depression scale show improvements in scores over time in all groups, although without significant differences between groups. Significant differences were observed in post hoc analyses without correction in all groups and times: in the FD group between PRE and PER (*p* = 0.002) and between PER and POST (*p* = 0.026); in the MD group between PRE and PER (*p* = 0.005) and PER and POST (*p* = 0.002); in the HAD group between PRE and PER (*p* = 0.008) and PER with POST (*p* = 0.027). However, no significant differences were detected in the Tukey and Bonferroni post hoc analyses except in the PRE and PER analyses of the FD group (Tukey post hoc *p* = 0.041) and PER with POST of the MD group (Tuckey post hoc *p* = 0.040).

Positive correlations were found between multiple variables: weight and BMI (*p* < 0.001), EAT-26 score and age (*p* = 0.006), EAT-26 DIET score and BMI (*p* < 0.001), EAT-26 ORAL CONTROL and age (*p* = 0.007). In addition, positive correlations were found between BSQ and the following variables: BMI (*p* = 0.020), EAT-26 score (*p* = 0.005), EAT-26 DIET (*p* < 0.001), EAT-26 BULIMIA (*p* < 0.001); also between POMS Cholera and the variables: EAT-26 DIET (*p* = 0.030) and POMS Tension (*p* < 0.001). More positive correlations were found between POMS Depression and the variables: POMS Anger (*p* = 0.008), POMS Tension (*p* < 0.001), BSQ score (*p* = 0.012), EAT-26 score (*p* < 0.001), EAT-26 DIET (*p* < 0.001), EAT-26 DIET (*p* < 0.001) and POMS Tension (*p* < 0.001), EAT-26 DIET (*p* < 0.001), EAT-26 BULIMIA (*p* = 0.018), EAT-26 ORAL CONTROL (*p* = 0.048); also between POMS Fatigue and the variables: POMS Tension (*p* = 0.023), POMS Cholera (*p* = 0.039) and Energy (*p* = 0.023).

Negative correlations were also found between the variables EAT-26 ORAL CONTROL and weight (*p* = 0.039), as well as POMS Vigour with the following variables: BSQ (*p* = 0.015), EAT-26 score (*p* = 0.015), EAT-26 DIET (*p* = 0.035) and EAT-26 BULIMIA (*p* = 0.015).

## 4. Discussion

The main purpose of this study was to assess the influence of different personalised dietary plans in professional female handball players with energy deficit on eating disorders, mood and body image.

So far, no research has examined the relationship between personalised dietary plans and these psychological parameters in young professional female handball players.

In professional athletes, disordered eating behaviours and ED are common [59]. These disorders are associated with perfectionism and compulsive behaviour during exercise, leading to injury and emotional distress that negatively affect performance [60]. Stress, depression and anxiety among other psychological factors contribute to disordered eating behaviour and LEA in athletes [61]. 

Adherence to a specific diet in female athletes may be associated with behaviour consistent with disordered eating. For example, ketogenic diets have appeared to be an ally in the recovery of eating disorders because of their ability to decrease anxiety, food cravings and addiction to ultra-processed foods [62,63,64,65]. However, a recent systematic review with meta-analyses positively linked ketogenic diets to anxiety and depression [66]. Research in athletes found that consumption of a low-carbohydrate diet (such as the ketogenic diet) negatively affected the mood of trained female cyclists compared to moderate and high-carbohydrate diets [67].

De Borja et al. [68] found that 68.5% of female athletes adhering to a diet tested positive on eating disorder-screening questionnaires [68]. In particular, athletes following a low-carbohydrate diet were more likely to report eating disorders than athletes without dietary restrictions [68]. This is consistent with the results found in this research, where all plans followed current recommendations for athletes and carbohydrate intake was high, and may therefore justify why no differences in EAT-26 scores were found after the intervention. Furthermore, it is well known that psychotherapy is required to improve the presence of ED-related symptomatology, which was not taken into account in the present intervention [25].

Psychonutritional education in young adults is also crucial for observing improvements in eating behaviour. Buddeberg-Fischer et al. [69] offered three health promotion lessons covering topics related to beauty ideals, body awareness, healthy eating behaviour and nutritional physiology. As in our research, differences in scores were found over time, but there were no significant differences between the control and intervention groups.

The prevalence of anxiety/depression-related mental disorder symptoms in professional team sports is high. Kilic et al. [7] found a prevalence of 26% in professional handball players and 16% in those who were retired [7]. These data are consistent with the high scores obtained on the POMS scale for depression at the beginning of the present investigation. The application of personalised dietary planning, whether FD, MD or HAD group, led to an improvement in these values over the 12 weeks of intervention. This is because there is a strong relationship between dietary factors and depression [70,71]. There is strong evidence that a healthy diet prevents the occurrence of depression, while a pro-inflammatory diet increases the incidence of depression [6,72,73].

The results found at the beginning of the research on the POMS scale are related to those of Mon-López et al. [74] in female indoor handball players. Our sample of young female players showed equal results on the Fatigue and Vigour scales, whereas the results were lower on the Depression, Tension and Anger scales. This can be explained by the fact that the measurements by Mon-López et al. [74] were conducted in the months after the global COVID-19 pandemic.

Furthermore, Jeitler et al. [75] in recent research showed that lifestyle interventions have a positive impact on quality of life and psychological parameters in patients with metabolic syndrome. At week 12, most self-reported outcomes improved in both groups, with no significant differences found between the different interventions but over time. The improvement in mood through the application of the Mediterranean diet has also been observed in research carried out by Wade et al. [76], where significant differences were shown in stress, depression, anger and confusion factors. The results of these investigations coincide with and support those found in the present study.

This research is the first to explore body image in professional female handball players; 20% of the players scored high on the BSQ scale, so it should be a factor to take into account when dealing with these athletes, especially because of its correlation with the presence of eating and mood disorders [77]. This value is significantly higher than that found in other research conducted in team sports. Specifically, Baldó Vela et al. [20] obtained scores of 4% for the BSQ, which can be explained by the fact that the sample was male rather than femal; moreover, their sample did not have a poor energy intake and had a lower prevalence of possible presence of ED [20]. Smith et al. [78] also found a high prevalence of body image dissatisfaction among the young cheerleaders and a relationship between body dissatisfaction and their coaches’ claims that they were physically smaller.

Despite the evidence supporting the Mediterranean diet and its benefits for certain organ systems, no significant differences have been found with respect to other well-prepared diets in athletes [23]. This coincides with our results, where the different diets showed the same effects over time on these cognitive parameters.

The variability of these data coincides with that found in other research in handball players; therefore, individual variations must be considered when designing psychonutritional programmes [4].

The main limitation of this research is the number of the sample; despite having all the national professional handball players of reference, it may be insufficient for significant results to be observed. The absence of meaningful results between the groups may be explained by the need to follow dietary planning for longer to give the markers a chance to show significant improvements.

The authors acknowledge that psychological factors are a complex field of research, and the results presented in this study should be interpreted with caution, given the diversity of individual responses. Future studies should be conducted in a larger group and with a longer measurement period in order to observe changes in all variables and differences between diets.

## 5. Conclusions

In conclusion, following different personalised dietary-nutritional plans based on current recommendations for athletes leads to improvements in mood and body image in young female professional handball players with low energy availability. No significant differences were found between following a free diet, a Mediterranean diet or an antioxidant-rich diet; however, significant differences were found over time following the different personalised diets. Longer interventions are needed to obtain significant improvements in eating behaviour variables.

## Figures and Tables

**Table 1:** Characteristics of female handball players (mean and standard deviation).

	FD (n = 7)	MD (n = 7)	HAD (n = 7)
**Age (years)**	22 ± 4	21 ± 3	22 ± 4
**Height (cm)**	171.0 ± 6.0	171.0 ± 7.2	173.0 ± 2.8
**Weight (kg)**	64.4 ± 5.1	70.5 ± 7.0	70.3 ± 6.7

FD: free diet group; MD: Mediterranean diet experimental group; HAD: high-antioxidant diet experimental group; cm: centimetres; kg: kilograms; mm: millimetres.

**Table 2:** Eating Attitude Test variables before, during and after the intervention in the free diet, Mediterranean diet and antioxidant-rich diet groups.

	FREE DIET (n = 7)						MEDITERRANEAN DIET (n = 7)						HIGH-ANTIOXIDANT DIET (n = 7)						Effect Time			Effect Time × Group		
	PRE		PER		POST		PRE		PER		POST		PRE		PER		POST		F	*p*	η²_p_	F	*p*	η²_p_
	X^¯^	SD	X^¯^	SD	X^¯^	SD	X^¯^	SD	X^¯^	SD	X^¯^	SD	X^¯^	SD	X^¯^	SD	X^¯^	SD						
**EAT-26 Total**	7.14	6.12	9.29	11.5	5.00	6.32	6.29	8.73	6.14	7.29	6.83	11.7	7.86	6.01	8.57	7.89	9.86	9.06	0.357	0.703	0.023	0.752	0.564	0.091
**Subscale Diet**	3.86	4.10	5.43	6.32	2.43	3.51	4.14	5.64	4.57	4.96	3.57	7.74	4.29	3.82	5.14	5.27	5.43	5.65	2.114	0.136	0.111	0.848	0.505	0.091
**Subscale Bulimia**	1.14	1.57	1.43	2.57	0.286	0.488	1.00	1.83	0.857	1.86	1.29	2.63	2.14	2.48	2.14	2.67	1.86	2.34	1.910	0.164	0.114	1.09	0.378	0.114
**Subscale Oral Control**	2.14	1.57	2.43	3.10	1.57	3.36	1.14	1.46	0.71	0.76	1.00	1.53	1.43	3.36	1.29	1.80	2.57	3.55	1.690	0.200	0.090	1.44	0.241	0.145
**Item 1**	0.29	0.76	0.71	1.25	0.33	0.82	0.71	0.76	1.14	1.21	0.83	1.33	0.86	1.21	0.86	1.21	1.00	1.29	0.137	0.872	0.009	0.206	0.933	0.027
**Item 2**	0.29	0.49	0.14	0.38	0.00	0.00	0.29	0.49	0.00	0.00	0.50	0.84	0.00	0.00	0.00	0.00	0.23	0.76	2.240	0.124	0.130	0.688	0.606	0.084
**Item 3**	0.67	0.98	0.86	1.21	0.33	0.52	0.43	0.79	0.57	1.13	0.50	0.84	1.00	0.82	0.86	0.90	0.86	0.90	2.200	0.128	0.128	1.160	0.347	0.134
**Item 4**	0.14	0.38	0.14	0.38	0.00	0.00	0.43	0.79	0.29	0.76	0.50	0.84	0.57	0.98	0.71	1.25	0.57	1.13	0.134	0.876	0.009	1.760	0.163	0.190
**Item 5**	0.29	0.76	0.43	0.79	0.00	0.00	0.57	1.13	0.49	0.79	0.17	0.41	0.00	0.00	0.29	0.76	0.29	0.76	0.625	0.542	0.040	0.748	0.567	0.091
**Item 6**	0.14	0.38	0.57	1.13	0.17	0.41	0.57	0.54	0.57	0.98	0.00	0.00	0.29	0.76	0.57	0.79	0.29	0.49	0.022	0.979	0.001	13.244	0.284	0.150
**Item 7**	0.00	0.00	0.00	0.00	0.00	0.00	0.00	0.00	0.00	0.00	0.00	0.00	0.14	0.38	0.00	0.00	0.00	0.00	0.028	0.973	0.002	0.667	0.620	0.082
**Item 8**	0.00	0.00	0.00	0.00	0.00	0.00	0.00	0.00	0.00	0.00	0.00	0.00	0.43	1.13	0.14	0.38	0.43	1.13	8.190	0.001	0.353	2.620	0.055	0.259
**Item 9**	0.00	0.00	0.00	0.00	0.00	0.00	0.00	0.00	0.00	0.00	0.00	0.00	0.00	0.00	0.14	0.38	0.00	0.00	0.205	0.816	0.014	0.595	0.669	0.074
**Item 10**	0.00	0.00	0.00	0.00	0.00	0.00	0.29	0.76	0.14	0.38	0.33	0.82	0.29	0.76	0.14	0.38	0.14	0.38	0.057	0.945	0.004	11.319	0.360	0.131
**Item 11**	0.29	0.76	0.71	0.95	0.33	0.82	0.43	0.79	0.29	0.76	0.67	1.21	0.00	0.00	0.43	1.13	0.43	1.13	0.086	0.918	0.006	10.578	0.394	0.1244
**Item 12**	0.43	0.79	0.71	1.11	0.17	0.41	0.29	0.78	0.43	0.79	0.33	0.82	0.00	0.00	0.00	0.00	0.14	0.38	1.960	0.159	0.115	1.080	0.382	0.126
**Item 13**	0.29	0.49	0.29	0.76	0.33	0.82	0.00	0.00	0.00	0.00	0.00	0.00	0.43	1.13	0.43	1.13	0.43	1.13	3.624	0.039	0.195	0.602	0.664	0.074
**Item 14**	0.57	0.98	0.29	0.76	0.17	0.41	0.57	0.98	0.43	0.79	0.50	1.22	0.57	1.13	0.29	0.49	0.14	0.39	0.749	0.482	0.048	0.609	0.659	0.075
**Item 15**	0.57	0.98	0.71	1.25	0.67	1.21	0.00	0.00	0.00	0.00	0.00	0.00	0.00	0.00	0.00	0.00	0.29	0.76	0.520	0.600	0.034	0.633	0.643	0.078
**Item 16**	0.00	0.00	0.29	0.76	0.17	0.41	0.00	0.00	0.43	0.79	0.00	0.00	0.14	0.38	0.71	1.11	0.43	1.13	4.204	0.025	0.219	0.770	0.553	0.093
**Item 17**	0.71	0.95	0.57	0.98	0.67	0.82	0.14	0.38	0.00	0.00	0.00	0.00	0.29	0.49	0.29	0.76	0.57	0.79	0.162	0.851	0.011	0.883	0.486	0.105
**Item 18**	0.43	0.79	0.14	0.38	0.00	0.00	0.00	0.00	0.00	0.00	0.33	0.82	0.00	0.00	0.14	0.38	0.14	0.38	0.271	0.764	0.018	0.863	0.497	0.103
**Item 19**	0.71	1.25	0.86	1.21	0.50	0.84	0.29	0.49	0.29	0.49	0.50	0.55	0.14	0.38	0.29	0.49	0.43	0.79	3.496	0.043	0.189	0.312	0.868	0.040
**Item 20**	0.00	0.00	0.00	0.00	0.33	0.82	0.00	0.00	0.00	0.00	0.00	0.00	0.43	1.13	0.14	0.39	0.43	1.13	12.400	<.001	0.452	1.600	0.201	0.175
**Item 21**	0.00	0.00	0.29	0.76	0.00	0.00	0.14	0.38	0.00	0.00	0.17	0.41	0.43	0.79	0.29	0.49	0.29	0.76	0.189	0.828	0.012	0.420	0.793	0.053
**Item 22**	0.29	0.49	0.14	0.38	0.00	0.00	0.29	0.76	0.29	0.76	0.50	1.22	1.00	1.41	0.86	1.07	1.00	1.29	0.065	0.937	0.004	0.834	0.514	0.100
**Item 23**	0.43	0.54	0.86	1.21	0.33	0.82	0.43	1.13	0.57	0.69	0.50	0.84	0.71	0.76	0.86	0.90	0.57	0.79	0.513	0.604	0.033	0.153	0.960	0.020
**Item 24**	0.00	0.00	0.00	0.00	0.17	0.41	0.14	0.39	0.14	0.39	0.50	1.22	0.00	0.00	0.00	0.00	0.29	0.76	0.127	0.881	0.008	0.050	0.995	0.007
**Item 25**	0.71	0.95	0.57	0.79	0.33	0.52	0.29	0.49	0.14	0.38	0.00	0.00	0.00	0.00	0.14	0.38	0.43	1.13	1.104	0.345	0.069	0.975	0.436	0.115
**Item 26**	0.00	0.00	0.00	0.00	0.00	0.00	0.00	0.00	0.00	0.00	0.00	0.00	0.14	0.38	0.00	0.00	0.00	0.00	0.205	0.816	0.014	0.595	0.669	0.074

EAT: Eating Attitude Test; BSQ: Body Shape Questionnaire; POMS: Profile of Mood States; **X¯**: Mean; SD: standard deviation; pre: before intervention; per: during intervention; post: after intervention.

**Table 3:** Body image and mood variables before, during and after the intervention in the free diet, Mediterranean diet and antioxidant-rich diet groups.

	FREE DIET (n = 7)						MEDITERRANEAN DIET (n = 7)						HIGH-ANTIOXIDANT DIET (n = 7)						Effect Time			Effect Time × Group		
	PRE		PER		POST		PRE		PER		POST		PRE		PER		POST		F	*p*	η²_p_	F	*p*	η²_p_
	X^¯^	SD	X^¯^	SD	X^¯^	SD	X^¯^	SD	X^¯^	SD	X^¯^	SD	X^¯^	SD	X^¯^	SD	X^¯^	SD						
**BSQ**	82.4	46.5	67.0	23.4	60.5	23.3	74.7	37.7	76.0	37.2	72.3	35.2	70.1	34.7	68.9	37.6	68.7	41.1	1.051	0.362	0.065	1.389	0.261	0.156
**Tension (POMS)**	0.71	0.44	0.87	0.26	0.77	0.31	0.67	0.32	0.83	0.15	0.63	0.38	0.66	0.24	0.87	0.23	0.56	0.32	0.642	0.637	0.079	1.389	0.265	0.085
**Vigour (POMS)**	1.21	0.37	1.19	0.41	1.08	0.63	1.17	0.36	1.37	0.47	1.35	0.25	1.07	0.27	1.07	0.38	1.01	0.47	0.288	0.883	0.037	0.869	0.430	0.055
**Anger (POMS)**	0.56	0.61	0.80	0.08	0.68	0.56	0.54	0.49	0.80	0.14	0.35	0.32	0.74	0.70	1.14	0.60	0.53	0.35	0.485	0.747	0.061	2.929	0.069	0.163
**Depression (POMS)**	0.39	0.45	0.74	0.27	0.42	0.34	0.34	0.42	0.60	0.15	0.18	0.25	0.26	0.15	0.61	0.30	0.31	0.45	0.404	0.805	0.051	1.244	0.303	0.077
**Fatigue (POMS)**	0.83	0.46	0.93	0.48	0.77	0.51	0.67	0.32	0.80	0.30	0.60	0.39	0.71	0.31	0.90	0.54	0.66	0.37	0.153	0.960	0.020	2.070	0.144	0.121

EAT: Eating Attitude Test; BSQ: Body Shape Questionnaire; POMS: Profile of Mood States; **X^¯^**: Mean; SD: standard deviation; pre: before intervention; per: during intervention; post: after intervention.

## Data Availability

The data presented in this study is available on request from the corresponding author. The data are not publicly available due to is personal health information.
